# The best of HPB in *BJS Open* 2023

**DOI:** 10.1093/bjsopen/zrae005

**Published:** 2024-02-13

**Authors:** Giovanni Marchegiani

**Affiliations:** Editor

The 2023 *BJS Open*'s inaugural issue launched a randomized clinical trial delving into the perennial quandary of interrupted *versus* continuous sutures for biliary-enteric anastomosis^1^. There was no discernible advantage favouring interrupted sutures, as indicated by comparable bile leak rates (14.6% interrupted *versus* 10.3% continuous sutures; *P* = 0.738) and a longer operative time (approximately 10 min). Although the results aligned with the intuitive sentiments of many hepatopancreatobiliary (HPB) surgeons, their documentation ignited a robust interaction on X (previously known as Twitter), with 121 posts contributed by 111 engaged users. It is imperative to note that the study’s inclusion criteria required a common bile duct diameter of at least 7 mm, leaving the management of smaller bile ducts an unresolved query.

The narrative of the year unfolded with the release of a successful leading article contemplating the contentious use of large language models in surgical science^2^. The advent of ChatGPT in late 2022 democratized access to these models, raising a lively discussion on their diverse applications and associated risks. Beyond facilitating writing tasks such as drafting research protocols, manuscripts, and grant proposals, these tools have potential in data extraction and even clinical decision-making. Garnering over 20 citations, this article stood out as one of the most cited papers of the year.

According to the European Commission’s estimates disclosed in the European Cancer Information System, cancer incidence and mortality witnessed an upward trend in the European Union, with particular emphasis on pancreatic cancer. This prominence was mirrored in the 2023 *BJS Open* publications, where manuscripts on pancreatic diseases outgrew those centred on hepatobiliary diseases. The analysis of 1300 pancreatoduodenectomies for malignancy from the Recurrence After Whipple’s study underscored substantial postoperative morbidity (17%) and mortality (4%)^3^. A parallel Dutch observational study reinforced these findings, elucidating how major complications and organ failure detrimentally impacted disease-free and overall survival^4^. The effect of complications on survival was largely mediated by adjuvant chemotherapy emphasizing, once again, the pivotal role of preventing or mitigating postoperative complications to improve treatment of this hideous disease. A significant stride in this direction was made with the publication of the first radiomics-based fistula risk score^5^. With an area under the curve of 0.81 in the external validation cohort, similar to that of existing scores, this novel tool had the added value of relying solely on preoperative CT-derived data. This aspect is particularly relevant as the ability to predict fistula risk *a priori* may enhance preoperative patient counselling.

Although numerous commendable papers were also published on hepatobiliary topics throughout the year, a comprehensive discussion of each is beyond our present scope. As 2023 draws to a close, we extend heartfelt gratitude to all the authors for their invaluable contributions and to the peer reviewers whose dedication made this wealth of knowledge dissemination possible. Looking forward, we welcome a continued influx of high-quality submissions in the coming year, ensuring the sustained publication of first-tier evidence in the field of HPB surgery.

## References

[1] Seifert L , von Renesse J, Seifert AM, Sturm D, Meisterfeld R, Rahbari NN et al Interrupted *versus* continuous suture technique for biliary-enteric anastomosis: randomized clinical trial. BJS Open 2023;7:zrac163 10.1093/bjsopen/zrac163 PMC9891343 36723996

[2] Janssen BV , Kazemier G, Besselink MG. The use of ChatGPT and other large language models in surgical science. BJS Open 2023;7:zrad032 10.1093/bjsopen/zrad032 PMC10037421 36960954

[3] Russell TB , Labib PL, Denson J, Streeter A, Ausania F, Pando E et al Postoperative complications after pancreatoduodenectomy for malignancy: results from the Recurrence After Whipple’s (RAW) study. BJS Open 2023;7:zrad106 10.1093/bjsopen/zrad106 PMC10689345 38036696

[4] Henry AC , van Dongen JC, van Goor IWJM, Smits FJ, Nagelhout A, Besselink MG et al Impact of complications after resection of pancreatic cancer on disease recurrence and survival, and mediation effect of adjuvant chemotherapy: nationwide, observational cohort study. BJS Open 2023;7:zrac174 10.1093/bjsopen/zrac174 PMC10036251 36959099

[5] Ingwersen EW , Bereska JI, Balduzzi A, Janssen BV, Besselink MG, Kazemier G et al Radiomics preoperative-fistula risk score (RAD-FRS) for pancreatoduodenectomy: development and external validation. BJS Open 2023;7:zrad100 10.1093/bjsopen/zrad100 PMC10561131 37811791

